# One of Those Mischievous Bills

**Published:** 1891-05

**Authors:** 


BUFFALO MEDICAL AND SURGICAL JOURNAL

A MONTHLY REVIEW OF MEDICINE AND SURGERY.

*EDITORS:*

THOMAS LOTHROP, M. D. - - WM. WARREN POTTER, M. D.

All communications, whether of a literary or business character, should be addressed to the editors : 284 FRANKLIN STREET, BUFFALO, N. Y.

VOL. XXX. MAY, 1891. No. 10.

### Editorial.

### ONE OF THOSE MISCHIEVOUS BILLS.

IT WILL be remembered that in our "leader" in the April number we called attention to some pending legislation in this State, which was calculated to do harm to the reform in medical education that was so happily begun last year.

We are informed that the Student's Bill, introduced by Mr. Jacobs, providing for exemptions from the operations of the Law of 1890, has passed both houses of the Legislature, and is now in the hands of the Governor.

It is to be regretted that neither the Senate nor the Assembly felt willing to grant a hearing to the profession in opposition to this bill. Moreover, the Medical Society of Erie County took formal action in regard to this measure, and sent a request to each of the members from Erie county asking their coöperation with a view to its defeat. It seems strange that neither the individual nor combined efforts of our senator and five assemblymen were adequate to even obtain a hearing pending the passage of a bill so universally opposed by the medical profession in this county and throughout the State. It cannot be possible that these servants of the people allowed such an important piece of legislation to slip through by default! We prefer to believe that they made strong efforts to prevent such a calamity and were overpowered in their attempts.

As we stated before, the bill is now in the hands of the Governor. We are persuaded to believe that he will not sign such a vicious bill, but that he will interpose his prerogative to veto the same. It is not possible that an Executive would lend such powerful aid towards perfecting the law providing for State Medical

Examination and License as he did last year, and then with one stroke of his pen render it all nugatory or of little effect. We prefer to class Governor Hill as one of the staunch friends of medical reform in this State, and shall expect him to stand with his hands on the throttle to prevent any attempts to overthrow the good work that he and his co-laborers have erected. Had it not been for Governor Hill's steadfast interest in the principle involved we are sure that the law of last year would not have been perfected. We do not believe he will knock out in one round the magnificent structure which he participated in building. We regard him as too consistent a friend to higher standards of medical education, to strike such a blow at the efforts of good men who have labored with him in this good cause.

